# 

**Published:** 1888-01-07

**Authors:** 


### CONTENTS.

**"Something Attempted"**—A Retrospect - - - 243

**Words of Consolation.**—Chapter LXII. The Sufferings of Christ - - - - - 244

**The Limits and Influences of a Nurse's Vocation.** By R. BRUDENELL CARTER, F.R.C.S. - - - 245

**Scotch Physicians and Surgeons.**—Sir JAMES Y. SIMPSON 246

**The Doctor's Pulpit.**—Every-day Science - - 248

**Professional Notes.** By GEO W. POTTER, M.D. - - 249

**No es and News** - - - - - 250

**The Nursing Mirror.**—The National Pension Fund and the Registration of Nurses.—Misapprehensions *v.* Facts - 252

**unnotations.**—"As they Face the Billows."—Suffering Children.—Epilepsy and Responsibiilty - - - 253

**Christmas at the Hospitals.**—Guy's.—University College.—Central London Throat and Ear.—The South Infirmary, Cork 254

**Rachel Challice's Vow.** By the Author of "A Future on Trust," etc.—Chap. I. The Vow - - - - 255

**Hospital Management and Methods of Raising Income.** By W. A. WEBSTER - - - 257

**The Amelioration of the Present Condition of Midwives.**—By JAMES H. AVELING, M.D., F.S.A. - - 258

**The Case of Miss Buchanan** - - - - - 259

**Amusements with Profit for all Ages.**—Work Competition.—For Men and Women.—The Children's Corner, Results, etc., etc. - - - - - - 260

**NOTICE.—A SPECIAL SUPPLEMENT.—"THE HOSPITAL" ALMANAC, 1888, COMPILED BY F. C. CARR-GOMM, ESQ., CHAIRMAN OF THE LONDON HOSPITAL, IS PRESENTED WITH THIS WEEK'S ISSUE.**

